# Overall Quality of Fruits and Vegetables Products Affected by the Drying Processes with the Assistance of Vacuum-Microwaves

**DOI:** 10.3390/ijms18010071

**Published:** 2016-12-30

**Authors:** Adam Figiel, Anna Michalska

**Affiliations:** 1 Institute of Agricultural Engineering, Wrocław University of Environmental and Life Science, Str. Chełmońskiego 37a, 51-630 Wrocław, Poland; adam.figiel@up.wroc.pl; 2 Institute of Animal Reproduction and Food Research of the Polish Academy of Sciences, Division of Food Science, Str. Tuwima 10, 10-748 Olsztyn, Poland

**Keywords:** combined drying, vacuum-microwaves, osmotic dehydration, heat pump drying, quality, energy efficiency

## Abstract

The seasonality of fruits and vegetables makes it impossible to consume and use them throughout the year, thus numerous processing efforts have been made to offer an alternative to their fresh consumption and application. To prolong their availability on the market, drying has received special attention as currently this method is considered one of the most common ways for obtaining food and pharmaceutical products from natural sources. This paper demonstrates the weakness of common drying methods applied for fruits and vegetables and the possible ways to improve the quality using different drying techniques or their combination with an emphasis on the microwave energy. Particular attention has been drawn to the combined drying with the assistance of vacuum-microwaves. The quality of the dried products was ascribed by chemical properties including the content of polyphenols, antioxidant capacity and volatiles as well as physical parameters such as color, shrinkage, porosity and texture. Both these fields of quality classification were considered taking into account sensory attributes and energy aspects in the perspective of possible industrial applications. In conclusion, the most promising way for improving the quality of dried fruit and vegetable products is hybrid drying consisting of osmotic dehydration in concentrated fruit juices followed by heat pump drying and vacuum-microwave finish drying.

## 1. Introduction

Numerous studies showed that there is a link between the total intake of fruits and vegetables and the reduction of the risk of mortality from major age-related diseases including cardiovascular diseases, diabetes and cancer. Those beneficial properties are connected with the presence of substances that, if identified, might help in selecting products that could increase their consumption, thus obtaining major health improvements without drastically changing the diet [1]. Moreover, those natural biologically active components when properly handled might also be used as natural pharmaceutical products [2,3]. The seasonality of fruits and vegetables makes it impossible to consume and use them throughout the year, so numerous processing attempts have been made to offer an alternative to their fresh consumption and application. Processing has been a crucial aspect of the fruits and vegetables production chain that associates agricultural production with the delivery of food in the required form [4]. Besides increasing the usefulness and availability of processed groceries, processing is also aimed at optimizing nutrient availability and to maintain product quality along with the reduction of losses and wastes [5]. It also has a critical role in achieving foods and nutrition security of the products obtained [6]. It should be taken into account that the quality of the final products is strictly connected with the processing operations and conditions and might noticeably differ from natural non-processed commodities [7]. Among numerous possible effects of processing on the overall quality of fruit and vegetable products, the most important are loss/improvement of naturally occurring components, formation of novel constituents with an increased antioxidative potential or pro-oxidant activity and interactions between different compounds [7]. Thus, availability of phytochemicals and nutrients in processed products is strictly dependent on the methods applied for their handling and those products should be carefully chosen in order to design new products for specific purposes or to complete a balanced diet [8].

## 2. Drying Processes and Their Combinations Used for Fruit and Vegetable Preservation

Among the processes applied for fruit and vegetable preservation, drying has received special attention as this method is currently regarded as the most common way for obtaining food and pharmaceutical products. It mainly aims at extending the product and/or its constituent’s shelf life by preventing the growth of microorganisms [9]. There are numerous drying techniques offered to dehydrate different kinds of raw materials in order to obtain products of a high quality dependent on processing parameters [10]. However, only some have found broad application in the food and pharmaceutical industries. Among the techniques used, freeze-drying (FD) is considered as the best method because low-temperatures applied in this process allow the highest retention of bioactive compounds comparable with the raw material [11]. Sanchez et al. [12] indicated that encapsulated freeze-dried cherry juice in powdered form resulted in a higher stability of total monomeric anthocyanins during storage at 38 °C in comparison to the juice and its concentrate. For this reason, this process is often used for obtaining natural pharmaceutical products [13,14]. In the case of freeze-dried fruits and vegetables, such products are characterized by minimal shrinkage and low alterations concerning both the chemical composition and color [15]. It should be highlighted that a commercial freeze-drying process is often neither robust nor efficient, thus other methods are required to diminish the production costs. Spray drying (SD) is an effective technique in which liquid products are directly turned into powders. SD is used broadly in industry, especially for pharmaceuticals production and in processing of diary and fruit products [16,17]. This technique was found to be 4–5 times cheaper when compared to freeze-drying [18]. Moreover, it was proven that application of SD process for dehydration of chokeberry juice into powders resulted in better retention of total phenolic compounds, total flavonoids, total monomeric anthocyanins when compared to FD [19]; however, in the literature there are limited data connected with comparison of both drying techniques in terms of the changes of the biologically active compounds. 

In food processing, convective drying (CD) is still one of the most extensively used method for dehydration of food products [20]. During CD, a drying agent consist mainly of hot air that simultaneously provides the energy necessary for water evaporation and subsequently evacuates water vapor out of the dryer. Even though this method does not require large investment costs and is effective during the first stage of drying process, it has some disadvantages and limitations. The product quality is relatively low mainly due to shrinkage [21] that involves a hard texture [22]. Moreover, during convective drying, the oxidation processes caused by hot air results in significant chemical alterations in the profile and the content of the thermolabile biologically active compounds [23]. Overall, hot air drying leads to taste, color and nutritional deterioration of agricultural products [24]. It was shown that hot air drying might significantly decrease the quality of the products in terms of the content of total polyphenolic compounds and antioxidant capacity in comparison to sun- and shade-drying processes [25]. A prolonged duration of the process time and a great amount of energy crucial for obtaining products with low moisture content are the major disadvantages of CD [26]. From the practical point of view, those limitations might be minimized by introducing innovative technologies such as a flash drying [27] and impinging stream drying [28] which offer relatively huge transfer of heat and mass during dehydration process. Another possibility is an application of an ultrasound-assisted drying that might significantly reduce the drying time by structural changes that enhance water diffusion [29]. On the other side, it should be highlighted that those changes might have a negative effect on the texture of dried product. Another emerging technology used for food products dehydration is a pulsed fluidized bed drying [30]. More advanced method is a vacuum microwave drying (VMD) due to a significant reduction in the drying time and a relatively good quality of dehydrated product with a high energy efficiency [31]. The capital expenditures and high costs of electric power are not encouraging for application of only this method [32]. At the beginning of VMD, the intensive water evaporation from the raw material might exceed the capacity of the vacuum pump. Therefore, a reduction of the material weight subjected to drying or the application of a larger vacuum installation is required. This issue might be overcome by the pre-drying using the convective method. As a result of pre-drying, the mass loads of vacuum microwave equipment might be radically decreased [33]. The combination of pre-drying of raw materials by convective method before VM finishing drying (CPD-VMFD) may reduce the total cost of dehydration maintaining a high quality of the obtained product [34]. Additionally, properly applied VMFD may favor formation of biologically active constituents that were not present in the raw material [35], simultaneously providing the crispy texture of the final product [36]. The quality of a dried product can even be improved and the total cost of dehydration might be reduced by application of heat pomp pre-drying (HPPD) instead of CPD. Heat pomp pre-drying process enables high energy efficiency by latent heat recovery [37] and contributes in a higher retention of polyphenolic compounds content, antioxidant potential and acceptable appearance [38]. In terms of energy efficiency and product quality, better results may be obtained by using a superheated steam under vacuum as a drying agent instead of a hot air in the HPPD. Under these conditions, the contact with oxygen is reduced to the minimum and the heat exchange is significantly improved [39]. The chemical composition of the dried fruit and vegetables might be drastically changed in terms of bioactive compounds and antioxidant capacity by the osmotic dehydration in concentrated juices applied as a pre-treatment procedure before drying. During the osmotic pre-treatment that aims at a water loss, simultaneous solids gain plays a crucial role in mass exchange [40]. An optimal concentration of the osmotic solution assures a high effectiveness of dehydration and, apart from beneficial chemical fortification, improves the flavor of the dried product due to gained solids. It was proven that an ultrasound during osmotic pre-treatment might accelerate the process of mass transfer [41,42] and, therefore, might enhance positive chemical changes that appear in the material [43].

To summarize, a combination of traditional dehydration methods and novel ones might significantly improve the quality of the final products. However, dehydration is an energy consuming process regardless of the drying method applied. Therefore, different energy reduction methods and potential use of renewable energy should be considered when designing new drying techniques. On the other hand, the quality of the final products in terms of the presence of biologically active constituents is strictly connected with the techniques and parameters applied, thus both aspects should be taken into consideration during designing of final products.

## 3. The Effect of Microwave Vacuum Drying on the Overall Properties of the Dried Products

**Figure 1:**
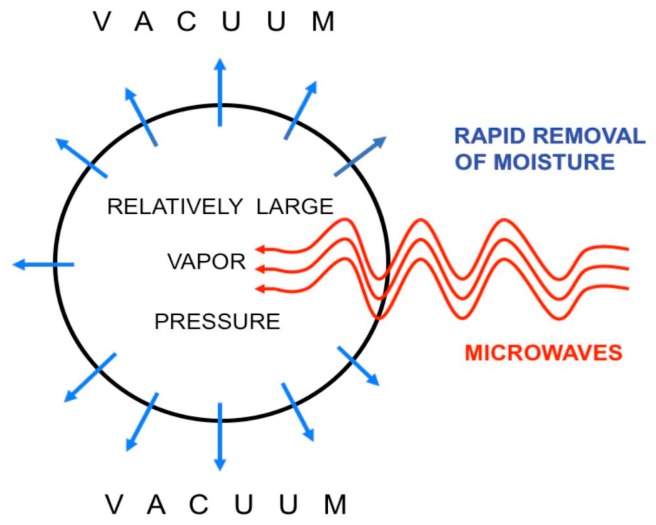
The vacuum-microwave drying process.

**Figure 2:**
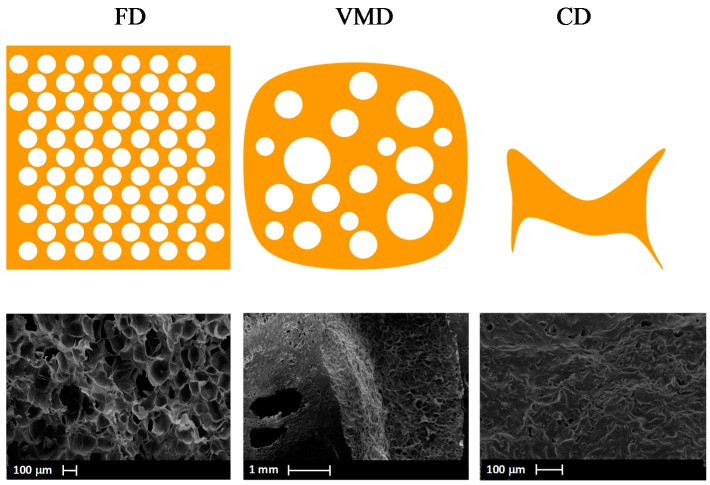
Structure of beetroot samples dehydrated by freeze-drying (FD), vacuum-microwave drying (VMD) and convective drying (CD).

**Figure 3:**
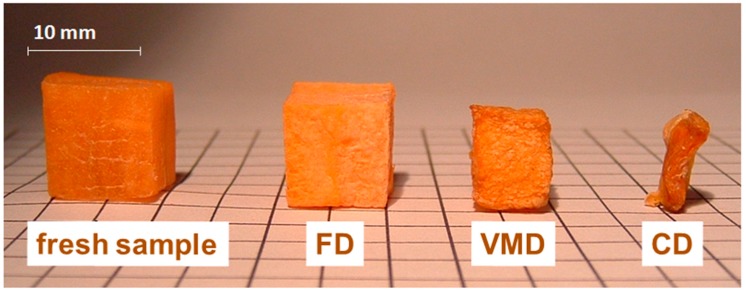
Shrinkage of carrot cubes dehydrated by freeze-drying (FD), vacuum-microwave drying (VMD) and convective drying (CD).

**Figure 4:**
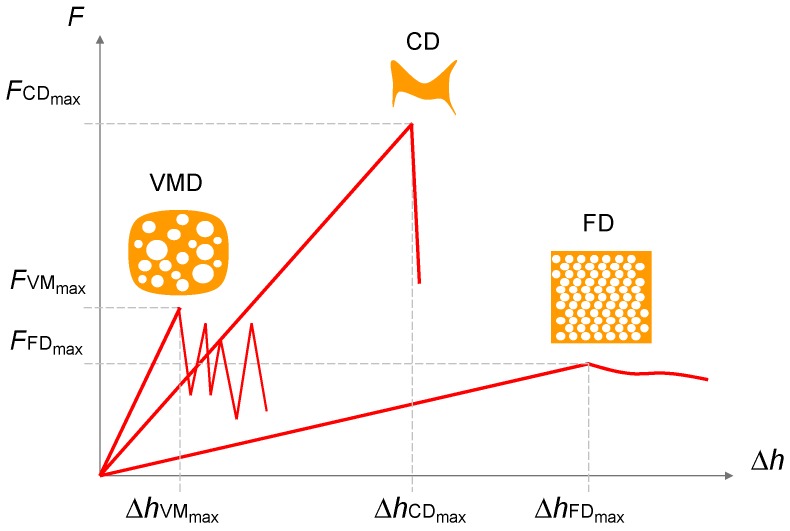
Typical force–deformation relationship *F* = f(Δ*h*) in compression test for products dehydrated by freeze drying (FD), vacuum-microwave drying (VMD) and convective drying (CD).

Among mentioned above drying techniques, a special attention has been drawn to the vacuum-microwave drying (VMD) due to its influence on the final quality of the products obtained. The VMD has already been successfully applied to reduce the moisture content of numerous plant materials, i.e., apples [36], blueberries [44], cranberries [45], strawberries [46], bananas [47], pumpkin [48], carrots [49], garlic [50], peanuts [51], ginger [52] and herbs [53]. During VMD, the energy of microwaves is absorbed by water dipoles located inside the processed material [54]. This creates a relatively high pressure in the center of the dried material and allows for a rapid transport of moisture to the vacuum preventing the material structure collapse (Figure 1). Due to this puffing phenomenon [55], the physical properties of dried material might be noticeably altered. The shrinkage of VMD products is significantly lower, the rate of drying is noticeably higher and the duration of the drying process is considerably shorter in comparison to convective drying (CD). Puffing phenomenon associated with a fast dehydration is responsible for a porous structure of VMD product; however, the number of pores is smaller and the size of pores is bigger when compared to freeze drying products (Figure 2). Consequently, the shrinkage of VMD product is a slightly higher than that of FD but significantly lower in comparison to CD product (Figure 3). The structural changes caused by the puffing influence the formation of a crispy texture of VMD products characterized by low values of force and deformation in the breaking point during compression test (Figure 4). For example, a compressive strength test indicated that the breaking force for VMD beetroot cubes dried at 480 W was 60% lower than for CD cubes dried at 60 °C [22] which confirmed positive changes owed to the puffing phenomenon that occurred in the structure of dried material. The structural changes in the dried product can be evaluated by rehydration test that lies on the determination of water absorption capacity (WAC), dry matter holding capacity (DHC) and rehydration ability (RA) [56]. Higher values of those indicators demonstrate better texture associated with the porous structure formed by puffing. The increase in microwave power from 240 to 720 W during MVD of apple slices increased WAC values from 0.67 to 0.77, DHC from 0.18 to 0.24 and RA from 0.13 to 0.19, whereas the values obtained for CD were, respectively, 0.58, 019 and 0.12 [57]. On the other hand, the increase in microwave power from 240 to 720 W enlarged the relative volume from 50% to 80% of VMD garlic parts due to lower shrinkage, which was confirmed by increased WAC values from 0.23 to 0.3 kg·kg^−1^ determined in humid air [58].

Application of VMD may significantly influence the chemical composition of the dried material. The study concerning dehydration of strawberries showed that VMD might produce the dried products with better retention of, e.g., anthocyanins, flavanols and ascorbic acid in comparison to CD [35]. The microwave vacuum drying process, when appropriately applied, may result in the quality of the final products comparable to freeze drying [55] being considered as one of the best drying method in terms of chemical composition of plant materials [52]. The proper application of VMD mainly concerns the control of microwave power, which is a crucial factor affecting the temperature of VMD material. On the one hand, the relatively high temperature usually degrades native bioactive compounds, but, on the other hand, contributes to the formation of new compounds with proven strong antioxidant capacity as products of the Maillard reaction/caramelization [59,60,61]. It was indicated that during VMD of plum halves, the increase in power from 120 W to 480 W almost doubled the content of hydroxymethylfurfural, a Maillard reaction/caramelization product, and a significant increase in antioxidant capacity measured by TEAC ABTS (Trolox Equivalent Antioxidant Capacity with application of ABTS^•+^ radical cations) and FRAP (Ferric Reducing Antioxidant Potential) methods was noted [62]. In order to achieve a favorable chemical composition that guarantees possibly the highest retention of natural biologically active components present in dried product, the temperature of VMD material should be kept at the safe level. An increasing temperature versus decreasing moisture content of dried material during VMD resulted from generation of heat energy by dipoles of water due to microwave radiation [54] reveals that at the very end of drying there is a critical point characterized by maximum temperature. Beyond this point, the temperature of dried material decreases because the generated energy is lower than the loss of energy that results from the water evaporation and heat flow arising from the gradient of temperature [22]. Understanding the temperature behavior of VMD material leads to proper application of reduced microwave power in an optimal way in terms of processing time and quality of the final product [63]. The effect of processing time and maximum temperature on different chemical parameters of selected plant materials is summarized in Table 1. In the case of strawberries [35], cherries [63], jujube [64] and plums [62], VMD resulted in comparable amount of polyphenolic compounds to freeze-drying process within the significantly shorter drying time, whereas similar level of volatile constituents after application of those drying methods was noted in marjoram [53]. This can be explained by the fact that the generation of heat during VMD might create a high vapor pressure inside the plant cells that, in turn, might cause a release of bioactive compounds bounded to the cell wall [65]. It should be highlighted that a significant increase of individual polyphenolic compound was indicated when VMD was used for plum dehydration. In this case, a noticeably higher content of 4-*p*-coumaroylquinic acid was noted after VMD when compared to FD [62]. Vvadenskaya et al. [66] reported that some of the individual flavonols are only present in cranberry products when processed what confirms that drying methods have a significant effect on the quality of the final products. Other studies proved that application of microwave vacuum drying resulted in the highest retention of anthocyanins being at the similar level as after FD when compared to hot air drying of raspberries [67] and cranberries [68].

It should be noted that, although vacuum microwave drying used for plant material dehydration is relatively fast and might ensure a quality of the dried products comparable to freeze-drying, it is not fully recommended for industrial applications as a single technique due to the investment expenditures and high costs of electric power necessary to supply magnetrons. Therefore, an option to use this fast dehydration technique is application of convective pre-drying (CPD) being more effective at the first stage of drying [33].

## 4. Convective Pre-Drying Followed by Vacuum-Microwave Finish Drying (CPD-VMFD)

**Figure 5:**
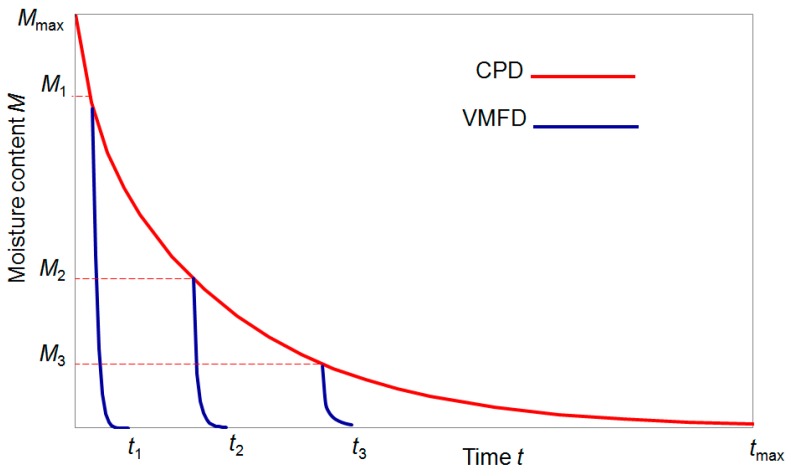
Drying kinetics for combined drying consisted in CPD until different levels of moisture contents followed by VMFD. *M*_1_, *M*_2_ and *M*_3_ represent a high, medium and low moisture contents obtained after CPD whereas *t*_1_, *t*_2_ and *t*_3_ denote total drying times of combined drying consisted of CPD until *M*_1_, *M*_2_ and *M*_3_ followed by VMFD.

High quality of the food product is an important aspect for industrial processing, but the final cost of production should be considered, as it might be a decisive factor for the success of the investment. For this reason, dehydration of plant materials by combined method consisting of convective pre-drying and vacuum-microwave finish drying (CPD-VMFD) is a compromise solution because it provides benefits of VMD at high productivity ensured by CD. It was proven that application of convective pre-drying of the material before VMFD reduced the total cost of dehydration and might improve the quality of dried tomatoes [69], nutritional value of strawberries evaluated in terms of ascorbic acids, anthocyanins content and antioxidant capacity [70] and might enhance the quality of beetroot cubes [22]. The main difficulty of CPD-VMFD concerns the time when CPD should be replaced by VMFD. As the main role of CPD lies on the maximal water removal from the row material with satisfactory drying rate, VMFD should start at a critical point of CPD when drying kinetics is losing its linear character for the exponential decreasing of moisture content in time characterized by decreasing drying rate [71]. The drying kinetics of combined drying consisting of CPD performed until different moisture levels were reached with finish drying completed by microwaves is presented in Figure 5. It was assumed that, despite the excellent energy efficiency during VMD [69], CPD should be continued until the texture of the dried material is soft enough to retain susceptibility of the puffing during VMFD to diminish the investment costs and the electric power consumption, even though the initial application of CPD method lowers the energy consumption, especially at a higher microwave power. It requires exclusive studies for particular plant materials with different texture characteristics that depend on the moisture content. The drying experiments on combined CPD-VMFD showed that, during VMFD, appropriate control of microwave power might enhance the drying rate of the process [57,72] and might result in a comparable level of bioactive compounds present in the dried material [62] or even higher [22] when compared to FD products. In the case of herbs, the composition of volatile compounds and the sensory attributes are the main quality aspects in a selection of the drying technique. Taking above factors into consideration, it was stated that the best method for preservation of rosemary was combination CPD-VMFD [73], while single VMD was found as the best method for dehydration of oregano herb [74]. These results proved that botanic morphology is a significant factor that should be taken into account while analyzing the suitability of plant material to the way in which vacuum-microwaves are applied during drying. Specifically, the loss of volatiles results from the temperature of the material and the time of drying. An increase in microwave power usually reduces duration of the process and at the same time enhances the increase in the temperature of the dried material [75]. During application of microwaves for plants with delicate structure, i.e., oregano, the water evaporate easily from the inside of the dried material under a relatively low inner pressure and thus low temperature. Therefore, VMD can be used from the very beginning until the very end of the drying process providing satisfactory retention of volatile compounds while drying e.g., basil [76] and thyme [77]. In the case of herbs with more dense morphology such as rosemary the particles of water are more bounded to the cellular system affecting the increase in the temperature under microwave radiation due to the higher inner pressure. Such increase can be reduced by introduction of CPD that might significantly diminish the initial amount of water and thus might shorten the time of microwaving during vacuum microwave finish drying. The time–temperature relationship that influence the retention of bioactive compounds during VMD or combined CPD-VMFD of plant materials was also discussed for cherries [63] and jujube [64] indicating that a raw material should be dried with a possibly high microwave power until a certain critical temperature level is obtained giving the reason for immediate reduction of the power to the value which guarantees the maintenance of a safe temperature of the dried material for providing the highest retention of bioactive compounds present in the final product i.e., total phenolics, anthocyanins, derivatives of quercetin and also the attractive color. The maintenance of microwave power at a sufficiently high level during VMFD might additionally provide energy benefits [34].

## 5. Heat Pumps for Better Quality and Energy Efficiency in Combined Drying with VMFD

Heat pump drying (HPD) may successfully replace the convective drying process before using VMFD for dehydration of plant materials. The major advantage of using the heat pumps is to provide a dehumidified air with a high ability for absorbing the water from the material despite the relatively low processing temperature [37]. Due to this low temperature, HPD is recommended for dehydration of sensitive materials as herbs [78,79], pharmaceuticals [80] and selected agricultural products [38,81,82]. Importantly, the HPD process noticeably save energy as heat pumps are classified as renewable sources of energy with a high efficiency due to recovery of latent heat of the water vaporization [37].

**Figure 6:**
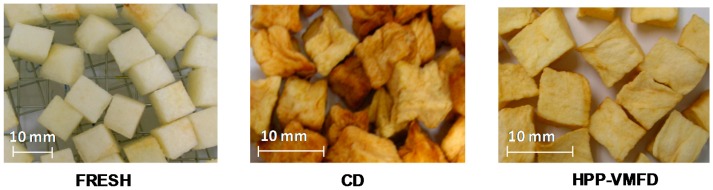
Appearance of apple cubes dehydrated by convective drying (CD) and vacuum microwave finish drying (HPPD-VMFD).

Combined HPPD-VMFD might provide dried products with a better appearance (Figure 6) and a higher content of total polyphenolic compounds (TPC) when compared to the other drying techniques. Chong et al. [83] showed that the TPC of HPPD-VMFD dried apple cubes reached 500 mg of Gallic acid equivalent (GAE) 100 g^−1^ dm, which was more than twice the TPC of HPD apple cubes. The higher content of TPC was mainly due to the significant reduction of the duration of the process caused by application of VMFD. Therefore, the entire process of heating treatment which reduce or halter the activity of enzymes that might degrade the phenolic compounds [84] was shortened and contributed to higher preservation of total phenolics that at the end affected the antioxidant capacity of the final products [85]. According to Donovan et al. [86] and Ferreira et al. [87], the phenolic compounds present in fresh fruits may be destroyed or converted to other constituents with stronger antioxidant properties. Such conversion might be an appropriate explanation for higher antioxidant capacity of convectively dried pears and papaya when compared to HPPD-VMFD samples [38]. The increased antioxidant capacity may also result from the formation of Maillard reaction/caramelization products, as was proven for plum products [88].

It was indicated that the content of total polyphenolic compounds present in fresh ciku (*Manilkara zapota*) [89] was decreased using the HPPD-VMFD to the level that was almost double when compared to samples obtained using CPD-VMFD and almost four times higher than after using single HPD [90].

## 6. Osmotic Dehydration as a Pre-Treatment before VMFD

The effect of drying time and temperature on the chemical composition of dried product is even more noticeable when osmotic dehydration (OD) is used as a pre-treatment. During osmotic dehydration of plant materials, three types of mass transfer occur simultaneously: (1) a water flux from the raw material to the osmotic solution; (2) a transfer of solids from the solution to the raw material; and (3) a migration of the natural solutes from the raw material to the solution [91]. Each type of mass transfer is likely to happen to some extent and it depends on the temperature, concentration, and the composition of the osmotic solution. OD of plant materials is performed in solutions prepared from sucrose or sodium chloride [92,93,94,95]. Previously, sucrose was applied for osmotic dehydration of strawberries [96], apples [97], pumpkin [98] and pepper [99], whereas NaCl solution was used for OD of tomato pomace [100], potato, carrot [101] and mushrooms [102]. Due to the high moisture content of agricultural products, OD is often applied as a pre-treatment procedure before finish drying aimed at obtaining final products with desirable taste and long shelf life [103].

Combined drying consisting of the osmotic dehydration and the vacuum microwave drying (OD-VMD) might additionally improve the physical properties, e.g., the texture of dried product. Application of OD using sucrose reduced the shrinkage and improved the rehydration capacity of vacuum microwave dried pineapple circular discs providing a softer texture and less hardened surface [104]. Moreover, a study on pumpkin slices showed that the optimal quality of dried product in terms of a cohesiveness, crispiness, appearance and taste can be achieved by application of the osmotic solution in concentration of 20%–40% of sucrose at the temperature 40 °C followed by a VMFD at microwave power 360 W [95]. The improved textural properties owned to a low cohesiveness and a high crispiness resulted from the lowest value of shrinkage (41%) obtained using a sucrose solution of concentration 40%. The same concentration of sucrose solution in osmotic pre-treatment guaranteed the lowest shrinkage of VMFD beetroot slices [105]. Torringa et al. [102] reported that the increase in concentration of the osmotic solution decreased the shrinkage of the mushroom finish dried by combined microwave-hot air drying method. This relationship can be explained by increased maximum temperature of VMFD material resulted from increasing of osmotic solution concentration [95,105]. The VM method ensures a lower shrinkage than traditional methods due to the puffing phenomenon [55] which is enhanced by a high inner pressure associated with the temperature of the dried material [22].

It can be presumed that application of NaCl might improve not only the taste of the dried products but also the dielectric properties of the material finish dried with microwaves [102]. It was indicated that pumpkin slices [106] and beetroot slices [105] did not required the pre-treatment in NaCl solution for improving the taste of the products but in terms of the texture of the dried material application of 15% NaCl solution was recommended. Moreover, an increase in NaCl concentration did not influence the rise of the temperature during VMFD, thus no improvement of drying rate was noted.

**Figure 7:**
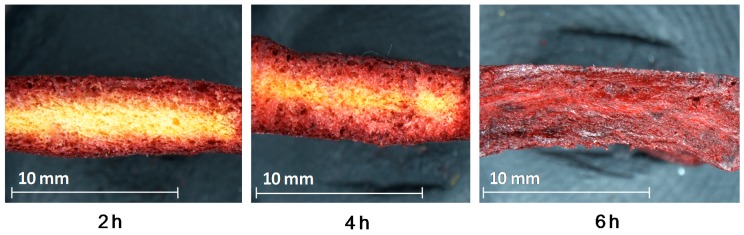
Cross-section of pumpkin slices pre-treated in concentrated chokeberry juice for 2, 4 and 6 h.

**Figure 8:**
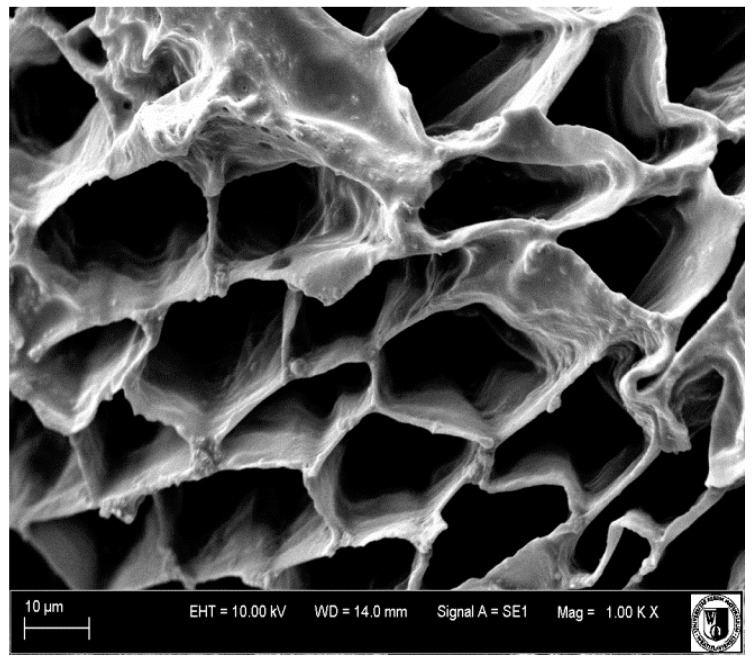
SEM (Scanning Electron Microscope) picture of beetroot sample dehydrated in concentrated chokeberry juice and VMFD at 480 W (1000× magnification).

Recently, in order to enrich dried plant materials in natural biologically active components, the application of OD solution consisted of concentrated fruit juices from, e.g., raspberry [107] or chokeberry [43], before microwave finish drying process was made [108]. Osmotic dehydration of pumpkin slices for 6 h in concentrated chokeberry juice resulted in 10 times increase of polyphenolics content using VMD in comparison to VMD pumpkin not subjected to OD. Such increase was caused by the replacement of water present in the fresh material by the concentrated juice containing more bioactive constituents in the osmotic solution [109]. The effect of water replacement by concentrated juice after 2, 4 and 6 h of OD is presented in Figure 7, whereas Figure 8 demonstrates capillaries of beetroot cellular system covered with the checkerberry juice concentrate. In comparison, a raspberry concentrated juice used as the osmotic solution increased five times the content of polyphenols in VMD pumpkin slices which was by 50% lower when compared to chokeberry juice [107].

Concentrated chokeberry juice was used for combined OD-VMFD applied for the beetroot dehydration. Results indicated that such OD solution before VMFD at 240 W resulted in three times higher content of total polyphenolic compounds and six times higher ability of samples to scavenge ABTS radical cations in comparison to fresh beetroot samples. Simultaneously, higher sensory notes were gain for OD samples in terms of texture, taste and smell of dried products than for non OD dried beetroots [43]. Osmotic solution consisted of concentrated apple juice before CD followed by VMFD caused a slight decrease of TPC in cherries [103]. A similar behavior may occur when dehydrated material demonstrates higher content of bioactive constituents than fruits used for preparing the osmotic solution. However, sensory benefits may be more important than a deterioration of chemical composition of valuable product especially when the alternative for improving the sensory attributes is connected with natural juices than a sucrose solution.

**Figure 9:**
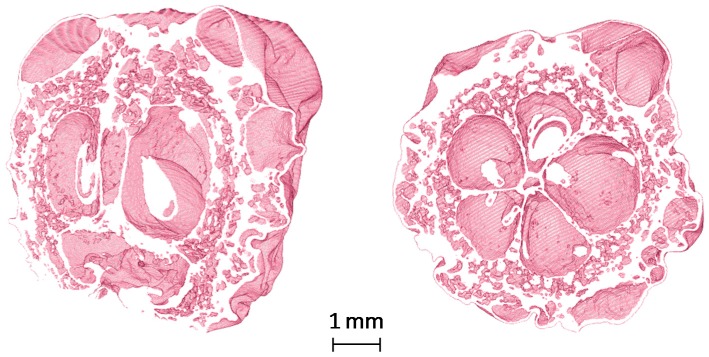
X-ray image of chokeberry fruit dehydrated by OD-CD-VMFD.

When physical properties are taken into account, the osmotic solution might be prepared from the same material as samples subjected to osmotic dehydration. This combination was applied for chokeberry products dried by hybrid consisted of OD-CD-VMFD (Figure 9) where the final products had increased crispiness due to the increase of temperature favoring larger porosity and total pore volume at lower final moisture content [110].

## 7. New Perspectives for Combined Drying as a Method of Preservation and Fortification of Dried Products

The combination of OD in concentrated fruit juices followed by CD and completed by VMFD can be successfully applied in the food processing on industrial scale. There is also a possibility of further improvement of this drying protocol by an implementation of the appropriate forms of each treatment. The osmotic dehydration might be assisted by the centrifugal forces [111] or supported by ultrasounds under lowered pressure in order to facilitate the process of mass exchange comprising water loss and solids gain [41,42]. Replacing traditional CD by HPD [37] with superheated steam as a drying agent under vacuum [39] creates conditions for higher energy efficiency [112] and enhances the retention of native bioactive phytochemicals due to lower processing temperature and diminishing the risk of oxidation processes [113]. Using of flash drying [27] or impinging drying [28] is also worth consideration as these drying techniques may significantly increase the productivity along with the shorten duration of the process. The appropriate use of the final VMFD still requires intensive studies including simulation and fuzzy optimization [114] due to the complex physical and chemical processes that might occur under the vacuum microwave process [115]. Such optimization might be aided by a computer imaging applied for in-line monitoring of the overall quality of product during drying processes [116,117,118]. A non-destructive computer-vision technology provides fast and efficient information about the physical state of the processed material that might improve the control of the food products during industrial processing [119]. The computer-vision system (CVS) was successively used to find the relationship between physical parameters (volume and color) and quality attributes (texture and moisture content), which helped to point the end of drying of apple slices [120]. A computer added image analysis might be also used for monitoring the physical, chemical, and sensory changes during storage of the dried agricultural products as those parameters are proven to be strongly correlated with the color characteristics of such materials [121,122,123].

## 8. Conclusions

Dehydration of agricultural products seems to be an extremely important matter, as currently the form and quality of food and pharmaceutical products decides their commercial success. Final quality in terms of physical and, more importantly, chemical properties of those products results of preparation steps and methods applied for their preparation. Recent studies concerning dehydration of agricultural products using vacuum-microwave technique showed that the most promising method is combined drying which ensures high quality at the lowest possible energy consumption. Osmotic dehydration in concentrated juices supply the products with additional biologically active constituents delivering them to the cellular system of the dried material, while the following heat pump drying, besides energy benefits, enables effective removal of the excess water at relatively low temperatures conducive to the retention of native compounds. During the final step of drying, an appropriate application of vacuum-microwaves with temperature control might contribute to release of native compounds from cellular structure or formation of new bioactive compounds as well as crispy texture of dried product. However, the optimization of the combined method of drying still requires additional studies comprising other forms of treatments enhancing water diffusivity, retention of valuable compounds and the process of fortification aimed at providing food products with excellent health-promoting properties and attractive sensory attributes.

## Figures and Tables

**Table 1:** Processing parameters (drying time (*t*) and maximum temperature (*T*)), total polyphenols content (TPC), antioxidant capacity (FRAP) and total volatiles content (TV) in different plant materials dehydrated by freeze drying (FD), convective drying (CD) and vacuum-microwave drying (VMD) at different processing parameters.

Plant Material	Parameter		*t* (min)	*T* (°C)	TPC (mg 100 g^−1^)	FRAP (mmol Trolox 100 g^−1^ dm)	TV (mg 100 g^−1^ dm)	References
Strawberries “Elsanta”	Fresh				2405.9	18.1		[35]
	FD			30	2411.5	16.8		
	CD	70 °C	550	70	1541.5	13.6		
	VMD	240 W	33		2302.4	14.4		
		360 W	21		2277.8	12.5		
		480 W	16		2253.2	16.2		
Cherries	Fresh				790.6	24.49		[63]
	FD			26	653.8	24.06		
	CD	50 °C	2400	50	518.2	16.62		
		60 °C	1450	60	488.6	13.26		
		70 °C	840	70	479.2	12.71		
	VMD	240 W	28	87.5	641.0	14.73		
		240/120 W	48	62.3	751.0	16.45		
		360/240 W	24	77.1	619.5	15.76		
		360/120 W	44	64.5	732.0	18.24		
		480/240 W	23	81.2	652.6	16.93		
		480/120 W	40	61.3	758.7	18.96		
Marjoram	Fresh						825	[53]
	CD	40 °C	2100	40			677	
		50 °C	500	50			637	
		60 °C	250	60			573	
	VMD	240 W	28	53			965	
		360 W	21	51			896	
		480 W	14	48			820	
Jujube “MSI”	FD			26	981	48.2		[64]
	CD	50 °C	660	50	430	31.7		
		60 °C	420	60	637	17.7		
		70 °C	330	70	367	15.9		
	VMD	120 W	96	74	813	27.1		
		480 W	28	142	567	26.2		
		480/120 W	64	79	982	47.8		
Plum halves	FD			20	1352.3	8.72		[62]
	CD	70 °C	3250	70	1135.9	6.73		
	VMD	120 W	120	75.8	1593.9	10.08		
		480 W	32	128.5	872.8	11.58		
		480/120 W	100	61.9	1064.5	10.31		
